# Mainstream Smart Home Technology–Based Intervention to Enhance Functional Independence in Individuals With Complex Physical Disabilities: Single-Group Pre-Post Feasibility Study

**DOI:** 10.2196/70855

**Published:** 2025-04-24

**Authors:** Dan Ding, Lindsey Morris, Gina Novario, Andrea Fairman, Kacey Roehrich, Palma Foschi Walko, Jessica Boateng

**Affiliations:** 1 Department of Rehabilitation Science and Technology School of Health and Rehabilitation Sciences University of Pittsburgh Pittsburgh, PA United States; 2 Department of Occupational Therapy College of Health and Wellness Johnson & Wales University Providence, RI United States

**Keywords:** physical disabilities, smart home technology, assistive technology, assistive technology service delivery, functional independence, participation, occupational therapy, artificial intelligence, AI

## Abstract

**Background:**

Mainstream smart home technologies (MSHTs), such as home automation devices and smart speakers, are becoming more powerful, affordable, and integrated into daily life. While not designed for individuals with disabilities, MSHT has the potential to serve as assistive technology to enhance their independence and participation.

**Objective:**

The study aims to describe a comprehensive MSHT-based intervention named ASSIST (Autonomy, Safety, and Social Integration via Smart Technologies) and evaluate its feasibility in enhancing the functional independence of individuals with complex physical disabilities.

**Methods:**

ASSIST is a time-limited intervention with a design based on the human activity assistive technology model, emphasizing client-centered goals and prioritizing individual needs. The intervention follows a structured assistive technology service delivery process that includes 2 assessment sessions to determine technology recommendations, installation and setup of the recommended technology, and up to 8 training sessions. An occupational therapist led the intervention, supported by a contractor and a technologist. Feasibility was evaluated through several measures: (1) the ASSIST Functional Performance Index, which quantifies the number of tasks transitioned from requiring assistance to independent completion and from higher levels of assistance or effort to lower levels; (2) pre- and postintervention measures of perceived task performance and satisfaction using a 10-point scale; (3) the number and types of tasks successfully addressed, along with the costs of devices and installation services; and (4) training effectiveness using the Goal Attainment Scale (GAS).

**Results:**

In total, 17 powered wheelchair users with complex physical disabilities completed the study with 100% session attendance. Across participants, 127 tasks were addressed, with 2 to 10 tasks at an average cost of US $3308 (SD US $1192) per participant. Of these tasks, 95 (74.8%) transitioned from requiring partial or complete assistance to independent completion, while 24 (18.9%) either improved from requiring complete to partial assistance or, if originally performed independently, required reduced effort. Only 8 (6.3%) tasks showed no changes. All training goals, except for 2, were achieved at or above the expected level, with a baseline average GAS score of 22.6 (SD 3.5) and a posttraining average GAS score of 77.2 (SD 4.5). Perceived task performance and satisfaction showed significant improvement, with performance score increasing from a baseline mean of 2.6 (SD 1.2) to 8.8 (SD 1.0; *P*<.001) and satisfaction score rising from an average of 2.9 (SD 1.3) to 9.0 (SD 0.9; *P*<.001).

**Conclusions:**

The ASSIST intervention demonstrated the immediate benefits of enhancing functional independence and satisfaction with MSHT among individuals with complex physical disabilities. While MSHT shows promise in addressing daily living needs at lower costs, barriers such as digital literacy, device setup, and caregiver involvement remain. Future work should focus on scalable models, caregiver engagement, and sustainable solutions for real-world implementation.

## Introduction

### Background

Assistive technology (AT) is federally defined as “any item, piece of equipment, or product system, whether acquired commercially, modified, or customized, that is used to increase, maintain, or improve functional capabilities of individuals with disabilities” [1]. Traditionally, AT refers to specialized devices designed for individuals with disabilities, such as wheelchairs, environmental control units (ECUs), and augmentative and alternative communication devices. Recent advancements in digital consumer electronics have expanded the scope of AT. Mainstream smart home technologies (MSHTs), such as home automation devices and smart speakers, have emerged as a viable alternative to traditional ECUs [2,3]. These technologies, while not explicitly designed for individuals with disabilities, can perform similar functions by promoting autonomy, reducing caregiver reliance, and enhancing quality of life [4-6]. Their affordability, accessibility, and integration of cutting-edge technologies, such as artificial intelligence (AI) and the Internet of Things (IoT), further underscore their potential. However, individuals with disabilities, particularly those who are not technology savvy, may struggle to envision how these technologies can meet their needs without proper guidance [2,4,5,7]. The wide variety of available options can also create confusion, especially when sales representatives lack training in addressing disability-specific needs or AT principles. Integration issues, such as ensuring MSHT devices work seamlessly with each other and existing AT while being compatible with the physical environment, further complicate their implementation. These challenges highlight the need for structured support to maximize the potential of MSHT as AT.

A scoping review by Cleland et al [8] identified 11 studies (up to July 2022) that examined the outcomes of home automation for individuals with disabilities. In total, 4 of these studies were noninterventional, focusing on exploring user experiences with their existing home automation technologies [4,9-11]. The remaining interventional studies primarily investigated specialized devices and applications or interventions using a room equipped with home automation technologies [12-17]. Notably, only 1 study specifically provided MSHT for individuals with amyotrophic lateral sclerosis, but it focused on a limited set of devices, including a smart speaker, light bulbs, and smart plugs [18]. Furthermore, only 4 studies were published after 2020 [4,13,14,18], emphasizing the need for updated research to reflect the rapid evolution of MSHT. A few recent studies after this scoping review have further explored the role of MSHT in supporting individuals with disabilities, highlighting both the potential and challenges associated with their implementation. Ripat et al [19] presented a case series of 3 individuals with high-level tetraplegia, emphasizing the benefits of low-cost, readily available technologies over stand-alone, dedicated devices. However, their effective use depends on appropriate access or interface methods, integration with current and future technologies, and the availability of training and maintenance support. Arthanat et al [7] developed an interventional protocol based on the human activity AT model for individuals with Alzheimer disease and their care partners, focusing on using MSHT to support personalized goals, such as safety, activity engagement, and caregiver connectivity [7]. While all 5 dyads reported positive goal attainment, challenges included unfamiliarity with MSHT, misconceptions about its use, and technological complexities that sometimes outweighed its benefits. These findings emphasize the need for comprehensive systems and support to optimize MSHT as AT solutions.

As MSHT continues to proliferate and integrate into everyday life, it is crucial to examine how these technologies can be effectively delivered to individuals with disabilities. While MSHT is readily available off the shelf, a structured AT service delivery process, commonly emphasized for traditional AT, may also be considered for MSHT [20]. AT services are defined as “any service that directly assists an individual with a disability in the selection, acquisition, or use of an assistive technology device” [1]. Research has shown that both the quality of AT and a robust service delivery process are vital to ensuring effective and tailored solutions for individuals with disabilities [21]. Our group conducted a qualitative interview study with 15 practitioners experienced in providing MSHT as AT. One of the key themes that emerged was the critical role of thorough needs and ability assessments before selecting devices. Practitioners noted that commercial technologies, despite their availability, still require specialized evaluation to ensure they meet the unique needs and abilities of clients with disabilities. Participants also highlighted the importance of demonstrating or trialing MSHT not only to aid in device selection but also to educate clients about its potential and secure their buy-in [3]. These insights align with the findings by Larsson Ranada and Lidström [22] that a structured service delivery process significantly contributes to the usability and satisfaction of AT devices.

Despite the growing availability of MSHT, limited research has systematically explored how these technologies can be effectively implemented as AT. Ripat et al [19] and Arthanat et al [7] briefly introduced their client-centered intervention protocols that addressed technology selection, customization, and training. The Delphi survey by Mun and Kim [23] identified 59 key features of a smart home modification program for individuals with physical disabilities, spanning 4 domains: user tasks, physical functions, environmental factors, and smart home characteristics. These features provide valuable guidance for implementing MSHT interventions, but further work is needed to explore how structured processes and individualized approaches can optimize MSHT’s role in supporting individuals with disabilities.

### Objectives

In this study, we developed and evaluated the feasibility of ASSIST (Autonomy, Safety, and Social Integration via Smart Technologies), a comprehensive MSHT-based intervention designed to enhance functional independence and participation among individuals with complex physical disabilities. Grounded in the principles of AT service delivery, ASSIST used a structured process to assess users’ needs, abilities, and contexts; select and acquire suitable devices; assist with installation and setup; and deliver targeted training. The intervention used a range of commercially available MSHT devices and mobile apps to address critical areas, including environmental control, activities of daily living (ADL), instrumental activities of daily living (IADL), communication, leisure, and emergency management.

## Methods

### ASSIST Intervention

#### Conceptual Framework

ASSIST was designed to enhance independent living and participation for individuals with complex physical disabilities through the use of MSHT. The intervention is guided by the human activity AT model [24], which comprises 4 key components: human, activity, AT, and context.

The activity component focuses on key activities that can be supported by MSHT based on user needs and priorities. Due to the dynamic and rapidly evolving nature of MSHT, there is often limited awareness among people with disabilities about the available technologies and their potential applications to meet diverse needs [2,3,5,7,20]. To address this gap, we developed and validated an ASSIST Functional Performance Index (AFPI) [2]. The AFPI features an item bank comprising 46 everyday tasks that can be addressed using off-the-shelf MSHT. These tasks span several areas, including environmental control, ADL, IADL, communication, leisure, and emergency management. The AFPI serves 2 primary purposes: helping clients identify and prioritize tasks most relevant to their needs and assessing task performance both before and after the intervention to measure the impact of MSHT.

The human component is considered an intrinsic enabler, emphasizing the importance of aligning individual abilities with the functional requirements of MSHT [25]. For the ASSIST intervention, a comprehensive assessment of human abilities is conducted to ensure an appropriate match. This includes evaluating motor, cognitive, visual, hearing, speech, and tactile abilities, as well as activity tolerance. In addition, factors critical for effective MSHT use, such as proficiency with mobile devices, existing technology use, and comfort and attitude toward technology, are assessed.

The context component encompasses cultural, physical, institutional, and social dimensions, all of which play a critical role in the selection and effective use of MSHT. Facilitators and barriers within these contexts are key considerations. For the ASSIST intervention, contextual factors, such as the home environment, type of caregiver support, and personal beliefs and values, are assessed.

The AT component is considered an extrinsic enabler. For ASSIST, this includes not only the MSHT itself but also access methods (eg, voice control, switches, and accessibility features), the integration of various technologies, and customization tailored to the client’s abilities and needs. In addition, “soft technologies,” such as training clients in the use of MSHT, are incorporated to ensure successful adoption and use.

#### Intervention Protocol

##### Overview

ASSIST uses a structured AT service delivery process, consisting of up to 10 in-person sessions (each 60-90 min) conducted in clients’ homes over a period of 3 to 6 months. A licensed occupational therapist (OT) leads the intervention and is supported by a technologist who assists in identifying and customizing technology solutions and a contractor who handles technology installation. The process includes 2 assessment sessions to identify technology solutions, followed by technology installation and up to 8 sessions for setup and training. The detailed components of ASSIST are outlined in the subsequent sections.

##### Intake

Before the assessment, clients were asked to provide background information on their health, functional status, insurance coverage, living conditions, caregiver support, and technology use, including their experience with traditional AT and MSHT, mobile device proficiency via the short-form Mobile Device Proficiency Questionnaire (MDPQ) [26], and their comfort and attitude toward technology.

##### Assessment 1

During this session, the OT introduced the ASSIST program and conducted a brief interview to clarify intake information as needed. The OT then worked with the client to sort and rank tasks using the AFPI, identifying the top 10 priorities. For each of these tasks, the OT collected self-reported baseline performance using the AFPI’s independence scale and had the client rate their perceived performance and satisfaction on a 10-point scale. In addition, the OT conducted functional assessments relevant to the identified tasks in areas such as upper extremity motor function, cognitive function, visual function, auditory function, tactile function, speech and language function, and activity tolerance. A mobile device assessment was also conducted, where the OT observed the client perform 5 smartphone tasks: making a phone call; sending an SMS text message; checking the local temperature using a weather application; using the internet to find the best local pizza place (with a distracting phone call); and downloading, moving, and deleting an application. Performance was documented regarding phone stabilization and positioning, manual access and control, speech and voice access, and cognitive considerations.

##### Assessment 2

This session aimed to help the client select smart home ecosystems and input methods that matched their functional abilities, comfort level, and use context to support task performance. The OT guided the client in trialing devices, input methods, and ecosystems using a custom smart home demo station and conducted an environmental assessment to ensure compatibility with the home’s physical characteristics and power sources. Others in the home were also consulted to understand their needs and preferences. The OT then collaborated with the technologist to identify technology solutions and shared the recommendations along with a training plan with the client.

##### Technology Installation

Once the client approved the technology selection and training plan, the OT coordinated the device acquisition and any necessary installation requiring hardwiring or significant physical labor by an affiliated contractor.

##### Training Sessions

Up to 8 training sessions were scheduled for each client. During each session, the OT began by assisting with pairing the devices and integrating them into the selected smart home ecosystems, if applicable. The OT then provided training on foundational digital skills, if needed, and familiarized clients with the MSHT devices designed to support the identified tasks. The Goal Attainment Scale (GAS) [27] was used to monitor progress throughout the training, which prioritized cognitive strategy training over traditional instruction-based methods. This approach taught clients to systematically plan, execute, and evaluate tasks, fostering critical thinking, adaptability, and self-reliance [28]. By emphasizing problem-solving, the training aimed to promote skill generalization and long-term use of MSHT, even in situations where devices malfunctioned, enhancing clients’ confidence and independence. In addition, clients were provided with a device manual in their preferred format (digital, print, or both). The manual included comprehensive information about the products, such as maintenance requirements and manufacturer contact details for troubleshooting and support. At the conclusion of the final training session, the OT collected self-reported posttraining performance using the AFPI’s independence scale and had the client rate their perceived performance and satisfaction on a 10-point scale.

##### Follow-Up

The OT conducted phone follow-ups at the end of the first and second months after the training to address any client questions, assist with troubleshooting, and provide additional training if needed. During the final in-person follow-up at 3 months, the OT collected self-reported performance using the AFPI’s independence scale, gathered client ratings of perceived performance and satisfaction on a 10-point scale, and conducted an exit interview.

### Feasibility Evaluation

#### Participants

Participants were eligible for inclusion if they met the following criteria: (1) aged ≥21 years; (2) had a physical disability that prevented independent control or access to their environment; (3) used a powered wheelchair as their primary means of mobility for ≥40 hours per week; (4) had been living in their own residence for at least 6 months; (5) scored 12 or higher on the Mini Montreal Cognitive Assessment 5-Minute Brief Version or 18 or higher on the traditional Montreal Cognitive Assessment (using modified scoring for individuals with physical disabilities and omitting written tasks), indicating no more than mild cognitive impairment [29]; (6) used a smartphone, tablet, or augmentative and alternative communication device; (7) were receptive to using MSHT in their residence; and (8) had internet service in their residence. Exclusion criteria included (1) being medically unstable, defined as having been hospitalized >3 times in the previous 12 months and (2) planning to change residence within 1 year.

#### Study Design

**Figure 1:**
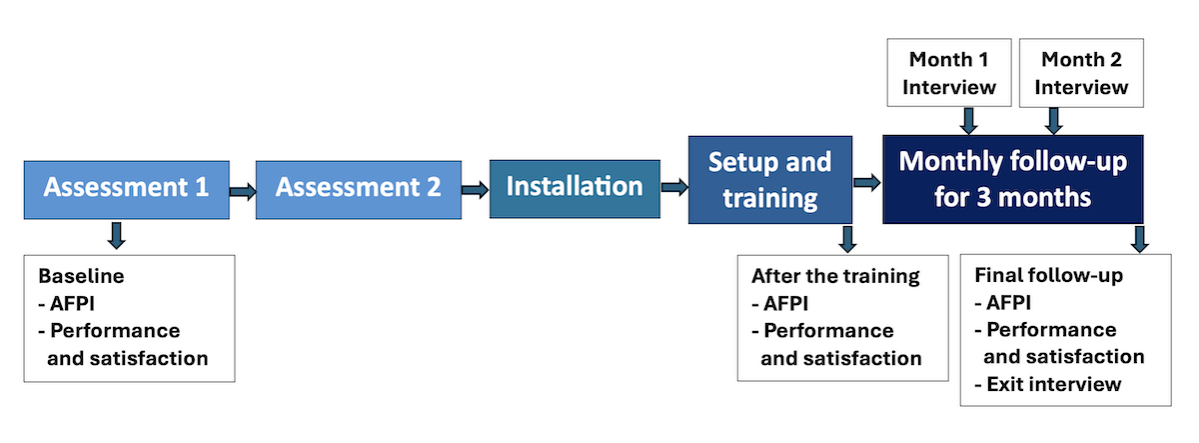
Study procedure. AFPI: Autonomy, Safety, and Social Integration via Smart Technologies Functional Performance Index.

The study used a mixed methods pre-post design, following the ASSIST protocol as previously described (Figure 1). After providing informed consent, participants completed demographic and intake questionnaires via REDCap (Research Electronic Data Capture; Vanderbilt University). Eligible participants collaborated with study OTs to identify up to 10 tasks to address. Each participant was allocated up to US $5000 to cover the costs of devices and installation services required for these tasks.

#### Data Collection

##### Technology Comfort and Attitude

Participants were asked to rate their technology comfort on a 4-point Likert scale with the following options: very comfortable, comfortable, uncomfortable, and very uncomfortable. They were also asked to select all descriptors they felt applied to their attitudes toward technology from a list, including satisfied, dissatisfied, enjoyment, frustration, confident, uncertain, simple, challenging, hesitant, excitement, fear, and overwhelming.

##### MDPQ-16 Tool

The 16-item short-form MDPQ is a validated tool designed to assess an individual’s proficiency with mobile devices across 8 key domains: mobile device basics, communication, data and file storage, internet, calendar, entertainment, privacy, and troubleshooting and software management [26]. Each item is rated on a 5-point scale with the following options: never tried (1), not at all (2), not very easily (3), somewhat easily (4), and very easily (5). Scores are averaged across the domains and summed to calculate a total proficiency measure, ranging from 8 (lowest proficiency) to 40 (highest proficiency).

##### AFPI Tool

The AFPI is a validated tool designed to evaluate an individual’s ability to perform everyday tasks that can be facilitated by MSHT to enhance performance and participation [2]. The AFPI comprises a 46-item task bank categorized into 6 domains: environmental control (9 tasks), ADL (5 tasks), IADL (20 tasks), communication and planning (6 tasks), leisure activities (4 tasks), and emergency management (2 tasks). Participants respond to a series of questions indicating how they typically complete each task and the level of assistance or effort required.

Their responses are coded on a scale from 1 to 11, as detailed in Table 1. While higher codes generally indicate greater independence, it is important to note that certain paired codes (ie, 2 and 3, 4 and 5, 6 and 7, 8 and 9, and 10 and 11) distinguish whether the individual uses technology for support or not, without implying a difference in overall independence.

**Table 1:** Autonomy, Safety, and Social Integration via Smart Technologies Functional Performance Index codes.

Code	Responses
1	Task not completed (due to lack of ability or resources) but has the desire to complete it
2	With complete assistance from another person and technology
3	With complete assistance from another person
4	With partial assistance from another person and technology
5	With partial assistance from another person
6	On my own with support from technology—hard
7	On my own without support from technology—hard
8	On my own with support from technology—some effort
9	On my own without support from technology—some effort
10	On my own with support from technology—easy
11	On my own without support from technology—easy

##### Self-Perceived Task Performance and Satisfaction

Participants were asked to rate their self-perceived task performance and satisfaction using a 10-point scale. For task performance, they were instructed to rate how well they currently complete the task, with 1 indicating “not able to do it at all” and 10 indicating “able to do it extremely well.” Similarly, for satisfaction, they were asked to rate their level of satisfaction with how they currently complete the task, with 1 indicating “not satisfied at all” and 10 indicating “very satisfied.”

##### Intervention Feasibility

The feasibility of the ASSIST intervention was evaluated across several key areas.

#### Participation and Attendance

This area evaluated the proportion of participants who completed the intervention and attended all scheduled sessions.

#### Tasks, Technology Solutions, and Costs

Tasks addressed covered the number of tasks successfully addressed compared with those that could not be addressed.

Categories of tasks included a detailed breakdown of the types of tasks addressed by the intervention (eg, environmental control, ADL, and IADL) and the specific tasks commonly addressed within each category.

Technology solutions included a classification of the types of technology solutions implemented to support the identified tasks.

Costs included the total costs incurred, including both technology costs and installation expenses.

#### Training Effectiveness

Participants’ progress in achieving their identified goals during the training sessions was assessed using GAS [27]. Participants collaborated with the study OTs to develop personalized goals based on the identified tasks. The OTs ensured that all goals adhered to the specific, measurable, achievable, relevant, and time-bound criteria [30]. For each goal, 5 levels of attainment were defined to measure progress. Level 0 indicated that the participant achieved the goal at the expected level. Positive levels, +1 and +2, reflected that the participant exceeded expectations, with +2 representing significant improvement beyond what was anticipated. Conversely, negative levels, –1 and –2, indicated outcomes that fell short of expectations, with –2 representing a much worse result than anticipated. At the beginning of the training, participants’ baseline levels were established, typically at –2 or –1. Progress was then reassessed periodically throughout the training sessions. The overall GAS score was computed at baseline and after the training using the following formula:



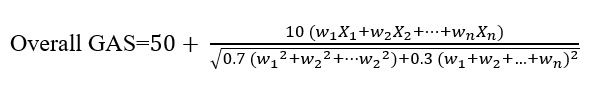



where *w* represents the weight assigned to each goal and *X* is the level achieved for each goal. In this study, as the goals were based on prioritized tasks, they were considered equally important, and equal weights were assigned.

#### Intervention Outcomes

Of the tasks addressed, the percentage that resulted in a shift from requiring assistance to achieving independence, the percentage that resulted in a change from complete to partial assistance or reduced effort if originally performed independently, and the percentage that resulted in no measurable change were covered in task outcomes.

Participant feedback included changes in self-perceived task performance and satisfaction between baseline and posttraining assessments.

#### Data Preparation and Analysis

All study data were retrieved from the study REDCap site. Descriptive analyses were conducted to summarize participant characteristics, tasks addressed, costs, GAS, AFPI performance, and self-perceived task performance and satisfaction. Changes in GAS, self-perceived task performance, and satisfaction before and after the intervention were analyzed using a paired sample 2-tailed *t* test or its nonparametric equivalent if assumptions were violated. All statistical analyses were performed using SPSS (version 28; IBM Corp).

#### Ethical Considerations

This study was approved by the Institutional Review Board at the University of Pittsburgh (STUDY19100361). All participants provided written informed consent before enrollment. All data were anonymized for analysis. Participants did not receive monetary compensation; instead, they were allowed to keep the technology devices recommended to them as part of the study.

## Results

### Overview

The study began in October 2021 with the enrollment of the first participant. From October 2021 to October 2022, the ASSIST protocol was trialed with 2 (11%) of the 18 participants to refine and streamline the process. Key adjustments included the addition of the MDPQ-16, self-perceived task performance and satisfaction measures on a 10-point scale, and finalizing the administration of the AFPI. Participants were asked to review the AFPI item bank to identify tasks they could not perform independently but wished to or tasks they found challenging or required assistance with. Then, they collaborated with the OT to prioritize tasks for intervention.

The remaining 16 (89%) of the 18 participants were enrolled starting in November 2022. One participant from this group withdrew after the first assessment session due to resistance to changes in their apartment or daily routine and a lack of readiness for the intervention. The remaining 15 participants completed the posttraining evaluation in December 2024. In total, 18 participants enrolled in the study. Of these, 17 (94%) completing the full study protocol and attending all scheduled sessions. As follow-up data collection is still ongoing, this study only reported results from baseline to posttraining evaluation. Results from follow-ups, monthly interviews, and exit interviews will be included in a future manuscript.

### Participant Characteristics

Demographic details for the 17 participants who completed the study are presented in Table 2. The participants in this study were aged between 23 and 80 years, with an average of 47.9 (SD 15.6) years. In total, 14 (82%) of the participants received paid assistance, either from professionals, family members, friends, or a combination of these, while 3 (18%) of them relied solely on unpaid caregivers. Weekly caregiving hours varied widely, ranging from 10 to 168 hours, with an average of 74.7 (SD 43.7) hours per week, highlighting the participants’ diverse support needs. All (n=17, 100%) participants had upper extremity motor deficits, and additional functional challenges were common. Some (n=7, 41%) participants reported visual challenges, with 6 (35%) retaining functional vision and 1 (6%) being legally blind. Some (n=7, 41%) participants experienced speech difficulties, and 1 (6%) reported auditory challenges. Moreover, some (n=8, 47%) participants faced sensory limitations.

All (17/17, 100%) participants used powered wheelchairs for mobility. Most controlled their wheelchairs using joysticks (15/17, 88%), while 12% (2/17) of the participants used alternative methods: chin control and head array. Smartphones were widely used among participants. In addition, 59% (10/17) of the participants owned iPhones, while 18% (3/17) of the participants used Google Pixel phones, and another 18% (3/17) used Samsung phones. Moreover, 6% (1/17) of the participants did not own a smartphone and used a family member’s phone when needed. For smartphone interaction, 47% (8/17) of the participants used a combination of touch, joystick, switch, or voice control; 35% (6/17) relied solely on touch or joystick; and 18% (3/17) exclusively used voice control. Beyond smartphones, 65% (11/17) of the participants had adopted at least 3 types of MSHT, 12% (2/17) had 1 or 2 pieces of MSHT, and 24% (4/17) participants did not own any MSHT products but possessed a tablet, a computer, or both.

**Table 2:** Participant demographics (N=17).

Demographic variables				Values
Age (y), mean (SD)				47.9 (15.6)
**Sex, n (%)**				
	Male		9 (53)	
	Female		8 (47)	
**Race^a^, n (%)**				
	Asian American		1 (6)	
	White		17 (100)	
**Ethnicity, n (%)**				
	Non-Hispanic		17 (100)	
**Education, n (%)**				
	High school diploma		2 (12)	
	Some college credit; no degree		3 (18)	
	Associate’s degree (eg, AA^b^ and AS^c^)		2 (12)	
	Bachelor’s degree (eg, BA^d^ and BS^e^)		6 (35)	
	Master’s degree (eg, MA^f^ and MS^g^)		2 (12)	
	Doctorate degree (eg, PhD^h^ and EdD^i^)		2 (12)	
**Primary diagnosis, n (%)**				
	**Cerebral palsy**		8 (47)	
		Quadriplegia spastic	5 (29)	
		Diplegia spastic	1 (6)	
		Hemiplegia spastic	1 (6)	
		Quadriplegic	1 (6)	
	**Cervical spinal cord injury**		7 (41)	
		C2^j^-C4^k^ complete (AIS^l^ A)	2 (12)	
		C2-C4 incomplete (AIS B or C)	2 (12)	
		C6^m^ incomplete (AIS B)	2 (12)	
		C4 (nontraumatic)	1 (6)	
	Multiple sclerosis (primary progressive)		1 (6)	
	Congenital myasthenic syndrome		1 (6)	
**Living situation, n (%)**				
	Alone		5 (29)	
	With others		12 (71)	
**Housing status, n (%)**				
	Own		14 (82)	
	Rent		3 (18)	
Medicaid home- and community-based services, n (%)				10 (59)
**Caregiving arrangement (some may select multiple options), n (%)**				
	Paid professionals		14 (82)	
	Paid family members or friends		6 (35)	
	Unpaid family members or friends		3 (18)	

^a^One participant reported both races.

^b^AA: associate of arts.

^c^AS: associate of science.

^d^BA: bachelor of arts.

^e^BS: bachelor of science.

^f^MA: master of arts.

^g^MS: master of science.

^h^PhD: doctor of philosophy.

^i^EdD: doctor of education.

^j^C2: the second cervical vertebra.

^k^C4: the fourth cervical vertebra.

^l^AIS: American Spinal Injury Association Impairment Scale.

^m^C6: the sixth cervical vertebra.

Participants reported varying levels of comfort with technology. Of the 17 participants, 1 (6%) described feeling very uncomfortable, while most felt more at ease, with 7 (41%) participants identifying as being very comfortable and 9 (53%) as being comfortable. In terms of attitudes toward technology, the most commonly chosen descriptors used by the participants included satisfied (n=10, 59%), frustration (n=9, 53%), confident (n=8, 47%), and enjoyment (n=7, 41%). Some participants described their experiences as challenging (n=6, 35%), uncertain (n=6, 35%), or overwhelming (n=4, 26%). Fewer participants chose terms such as dissatisfied (n=2, 12%) or fear (n=1, 6%). Some (n=5, 29%) participants selected only positive descriptors, 2 (12%) selected only negative descriptors, and 10 (59%) described a mix of positive and negative experiences.

Mobile device proficiency, as measured by the MDPQ-16, showed a wide range of abilities. The average score (excluding the first 2, 12% of the 17 participants whose data were not collected) was 29.8 (SD 9.5), with 3 participants scoring below 20, indicating significant challenges; 3 scoring between 20 and 30, reflecting moderate proficiency; and the remainder (n=9) scoring between 30 and 40, representing higher proficiency.

### Tasks, Technology Solutions, and Costs

#### Overview

Each participant was asked to identify up to 10 tasks, resulting in 155 tasks being identified (mean 9.1, SD 1.4 tasks per participant). Of these, 127 (81.9%) tasks were addressed (mean 7.5, SD 2.0 tasks per participant). Table 3 provides a detailed breakdown of the 127 tasks addressed across domains, including 64 (50.4%) for environmental control, 8 (6.3%) for ADLs, 37 (29.1%) for IADLs, 12 (9.4%) for emergency management, 3 (2.4%) for communication, and 3 (2.4%) for leisure.

In total, 28 (18.1%) of the 155 tasks were not addressed due to various factors, such as the absence of appropriate technology solutions, insufficient infrastructure, and the need to prioritize tasks for digital literacy training.

Some tasks could not be addressed due to the absence of appropriate technology solutions. For example, a smart shower controller was not implemented because the available products override manual controls, potentially leaving users unable to adjust the shower manually if the device malfunctions. Similarly, no suitable smart or standard scale was available for home weight monitoring for individuals who use powered wheelchairs. Side-to-side bed position adjustments were not possible with the smart bed frames currently on the market. In addition, while medication dispensers could help organize and dispense medications, they were not suitable for individuals with upper extremity motor impairments who could not reach and retrieve the medications independently.

Environmental constraints further posed challenges. For instance, the lack of nearby outlets prevented the installation of devices, such as bidets and air conditioners, in participants’ homes.

In other cases, tasks had to be reduced to allow participants sufficient time to improve their digital literacy and prevent them from feeling overwhelmed.

In terms of technology solutions, participants were first guided to select at least one smart home ecosystem to integrate their MSHT devices. A total of 6 (35%) of the 17 participants opted to use 2 smart home ecosystems, and the rest (11/17, 65%) chose a single ecosystem, as shown in Table 4.

A variety of MSHT devices were provided to address participants’ identified tasks. Products were selected based on compatibility with the participants’ chosen smart home ecosystems, the desired features, and environmental compatibility. These devices leveraged AI and IoT capabilities to enhance automation, remote control, and accessibility. An overview of the provided technologies and their functionalities is given in the subsequent sections.

**Table 3:** Tasks addressed (n=127).

Tasks addressed		Tasks, n (%)	
**Environmental**			
	Lock and unlock doors		11 (8.7)
	Control lights		10 (7.9)
	Answer doors		9 (7.1)
	Open and close doors		8 (6.3)
	Control fans		8 (6.3)
	Control window treatments		8 (6.3)
	View inside or outside for surveillance		5 (3.9)
	Adjust thermostat		4 (3.1)
	Building access		1 (0.8)
**ADLs^a^**			
	Bed mobility		4 (3.1)
	Clean oneself after toileting		2 (1.6)
	Hand washing		1 (0.8)
	Tooth brushing		1 (0.8)
Operate TV		3 (2.4)	
Emergency management		12 (9.4)	
Communication		4 (3.1)	
**IADLs^b^**			
	Vacuum or mop floors		6 (4.7)
	Charge technology devices		6 (4.7)
	Medication management		3 (2.4)
	Pet care		3 (2.4)
	Manage health care supplies		3 (2.4)
	Pay for purchase		3 (2.4)
	Shop in person		2 (1.6)
	Determine needed grocery or items		2 (1.6)
	Self-manage health		2 (1.6)
	Other tasks		6 (4.7)

^a^ADL: activities of daily living.

^b^IADL: instrumental activities of daily living.

**Table 4:** Smart home ecosystems chosen by study participants (N=17).

Smart home ecosystems	Participants, n (%)
Amazon Alexa	5 (29)
Google Home	3 (18)
Samsung SmartThings	2 (12)
Apple Home	1 (6)
Amazon Alexa and Google Home	4 (24)
Amazon Alexa and Apple Home	2 (12)

#### Lighting Control

Participants required flexible solutions for diverse lighting conditions, including ceiling lights, lamps with either standard or atypical bulbs, multiway switch configurations, and motion sensor controlled lights with extended illumination. The provided devices integrated IoT connectivity and AI-driven automation, allowing for application and voice control as well as automation tailored to user preferences and routines.

Smart in-wall dimmers and switches provided remote and automated lighting control while allowing manual operation without disrupting application or voice control. Companion switches for multiway installations maintained smart lighting functionality across multiple switch locations. Retrofit smart switches and button actuators provided remote control for nonsmart lighting fixtures without replacing existing wiring. Smart bulbs and plugs enhanced flexibility by enabling customizable lighting scenes and automation without modifying existing fixtures. Smart dimmer switches that were attached to existing light switches prevented accidental switch offs that would disable the functionality of smart light bulbs while allowing for local manual control.

#### Home Access

Participants received automated door and lock solutions that integrated with smart home ecosystems for enhanced safety and security. These devices leveraged AI-based access control, object recognition, and IoT-enabled remote operation, allowing for hands-free entry and real-time monitoring.

Automatic door openers with voice control modules or smart garage door openers enabled hands-free operation through voice assistants, allowing individuals with mobility limitations to open doors effortlessly.

Smart locks (retrofit and permanent solutions) provided keyless entry via mobile apps, biometric authentication, or voice commands, offering secure and customizable access.

Video doorbells (wired or battery-powered video doorbells with solar options) provided real-time video streaming, 2-way communication, and motion-triggered or object recognition–based notifications, with some models offering facial recognition to identify known visitors. When integrated with other smart home devices, video doorbells could trigger automated routines, such as turning on lights to enhance accessibility.

#### Window Treatment Control

AI-enhanced motorized solutions were provided to automate curtain and blind adjustments for improved accessibility. They could also automate adjustments based on time of day, room temperature, or sunlight exposure.

Retrofit curtain bots motorized existing curtain rods for voice and mobile app control. Retrofit options for tilt wand control adjusted blind slats via voice and mobile app control. Retrofit options for horizontal or vertical blinds or shades with continuous loop controls adjusted the blind tilt or lift or lowered the blind or shade via voice and mobile app control. Smart blinds (roller, cellular, and top down or bottom up) adjusted blinds via voice and mobile app control.

#### Climate Control

AI-enabled thermostats and fans automated temperature regulation, with some models featuring adaptive learning to adjust based on user routines.

Smart thermostats enabled temperature control via mobile apps or voice commands, with some models featuring occupancy sensors and learning algorithms for adaptive adjustments. Smart ceiling fans and standing fans provided scheduled automation and IoT integration to adjust airflow based on temperature sensors and voice or mobile app control. Smart bridges for existing fans converted nonsmart fans into IoT-compatible devices, enabling voice or mobile app control and automated control.

#### Surveillance and Inventory Management

Smart cameras, designed for indoor and outdoor use, provided surveillance and security, with some models featuring voice-controlled pan, tilt, and zoom functions and object recognition features. In addition, when paired with mobile apps such as Sortly (Sortly Inc), they helped participants manage health care supplies. When placed inside a refrigerator, they also facilitated the tracking of stored items.

#### Wearables for Emergency Support and Daily Assistance

Smartwatches, including Apple Watch (Apple Inc) and Google Pixel Watch (Google LLC), supported multiple tasks, such as accessing emergency services or contacts, making in-store payments, and managing personal health.

In addition, a variety of AI-driven and IoT-enabled solutions further supported participants’ independence and home automation. Textbox 1 provides a list of such solutions.

In terms of costs, all participants stayed within the US $5000 allowance. The overall cost of devices and installation services by the contractor averaged US $3308 (SD US $1192) per participant. Notably, 8 (47%) of the 17 participants selected “open or close door” as a desired task, which required relatively expensive automatic door openers that are not typically classified as MSHT. Excluding the cost of automatic door openers, the average cost per participant decreased to US $2234 (SD US $984).

**Solutions that supported participants’ independence and home automation.**

- Robotic vacuums: used artificial intelligence–powered navigation to optimize cleaning paths, avoid obstacles, and automatically lift mops based on floor type; features such as autoemptying bins and self-washing mop systems reduced maintenance needs
- Smart home hubs: centralized control for all Internet of Things–enabled devices, allowing seamless automation
- Smart button actuators: converted standard appliances into smart-enabled devices by automating button presses
- Smart power strips: enabled remote control of multiple devices with artificial intelligence–driven energy consumption tracking
- Smart smoke and CO detectors: provided real-time alerts and remote monitoring for safety
- Smart bed frames and voice-controlled bed systems: allowed users to adjust positions via voice commands or mobile apps
- Smart faucets and bidets: touchless control enhanced hygiene and accessibility
- Smart pet feeders and water fountains: enabled individuals to independently manage their pets’ feeding and hydration through automated schedules, with mobile app or voice control for portion adjustments and monitoring
- Streaming devices: provided access to digital media, enabling voice and mobile app control for seamless navigation of entertainment and news
- Smart Sensors (water leak sensors, contact sensors, etc): enhanced independence by providing real-time alerts for leaks, open doors or windows, or unauthorized access; these Internet of Things–enabled sensors could integrate with smart home systems for automated responses
- Essential Accessories: enhanced independent use of smart devices, including smartphone mounting systems for beds and wheelchairs, charging solutions for independent device charging, and mesh Wi-Fi systems to improve connectivity

### Training Effectiveness

**Figure 2:**
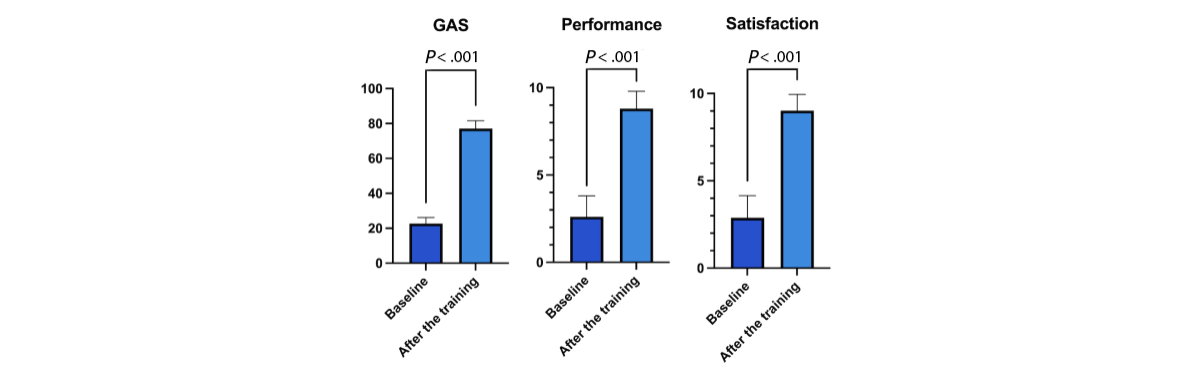
Baseline and posttraining comparisons. GAS: Goal Attainment Scale.

Participants completed an average of 5.6 (SD 1.7) training sessions. Working with the study OTs, they collaboratively established 117 goals, averaging 6.9 (SD 2.3) goals per participant. This number is slightly smaller than the total number of tasks identified, as some tasks were combined for efficiency, for example, combining “open or close doors” with “lock or unlock doors.” Nearly all goals (114/117, 97.4%) achieved at least level 0 (met expectations) on the GAS, and most (99/117, 84.6%) reached level 2 (significantly exceeded expectations). The average baseline GAS score of 22.6 (SD 3.5) increased to 77.2 (SD 4.5) after training (Figure 2), indicating substantial progress across participants. Paired sample *t* tests showed statistically significant improvements in GAS scores after training (t_16_=37.6; *P*<.001; Cohen *d*=6.0).

### Intervention Outcomes

On the basis of the AFPI results, 74.8% (95/127) of the tasks showed a shift from requiring assistance to achieving independence. An additional 18.9% (24/127) of the tasks demonstrated a transition, either from requiring complete assistance to partial assistance or, for tasks performed independently, a reduction in the effort required. Only 6.3% (8/127) of the tasks showed no measurable change.

Self-perceived task performance (excluding the first 2 participants whose data were not collected) improved significantly from a baseline mean of 2.6 (SD 1.2) to 8.8 (SD 1.0) after training, while satisfaction with task performance increased from an average of 2.9 (SD 1.3) to 9.0 (SD 0.9; Figure 2). Paired sample *t* tests indicated statistically significant improvements in both self-perceived performance (t_14_=13.8; *P*<.001; Cohen *d*=1.7) and satisfaction (t_14_=13.8, *P*<.001; Cohen *d*=1.7) after the intervention.

## Discussion

### Principal Findings

This study introduced ASSIST, a comprehensive intervention that leverages off-the-shelf MSHT to support individuals with complex physical disabilities. The findings demonstrated the intervention’s feasibility in improving functional performance and supporting independent living. Participants reported transitioning from relying on caregiver assistance to independently completing 74.8% (95/127) of their identified tasks, reducing the level of assistance or effort required for 18.9% (24/127) of the tasks, and significantly improving perceived task performance and satisfaction.

ASSIST uses a client-centered approach through 2 comprehensive assessment sessions aimed at identifying each individual’s unique needs, abilities, and context. This approach is essential not only because of the wide range of MSHT available and its potential to address numerous daily tasks but also because selecting the appropriate devices requires careful consideration of multiple factors, such as the user’s functional abilities, preferences, technology experience and comfort, and home environments. For example, we prioritized retrofit options for renters, provided mesh Wi-Fi systems to resolve connectivity issues, and selected devices with mobile apps that ensure accessibility for participants relying solely on voice control. We also addressed the concerns of others in the home. For example, parents of a participant were hesitant about installations in shared spaces, such as the living room and kitchen, fearing they would look “obvious.” After the OT showed them permanent solutions that matched their wall color, they approved. We also developed an environment checklist for different types of MSHT to streamline the selection process, ensuring compatibility and efficiency. Many of the factors addressed in ASSIST align with the 59 key features of a smart home modification program for individuals with physical disabilities identified in the Delphi survey by Mun and Kim [23]. This client-centered approach is further supported by the findings of Ripat et al [19], who emphasized the importance of addressing unique goals, circumstances, and contexts in their case series on the identification and assessment of electronic aids for daily living among individuals with high-level tetraplegia. Their work stressed the necessity of tailoring interventions to meet the distinct needs of each individual, even among those with similar injury levels.

The client-centered approach in ASSIST extends to its training sessions, where individualized plans are developed based on participants’ technology experience, comfort, attitudes, and mobile device proficiency. For example, 2 (12%) of the 17 participants with similar functional profiles, both relying exclusively on voice control for smartphones, had different technology attitudes and MDPQ-16 scores (16.5 vs 36). One described their attitude as “dissatisfied, frustrated, uncertain, and excited,” while the other chose “simple.” As a result, the study OT designed tailored plans, with the first participant starting with basic voice control training and the second covering advanced skills, such as creating routines. This approach aligns with Arthanat et al [7], who emphasized using GAS for individualized training in their MSHT intervention for individuals with Alzheimer disease and their care partners.

Notably, our client-centered approach was supported by several tools. First, many individuals are not familiar with or do not keep up with technological advancements. This issue was highlighted in our previous work on developing and validating the AFPI tool [2] and by Arthanat et al [7], who found that participants’ unfamiliarity with the technology was a major challenge in their MSHT intervention. Similarly, a UK policy report based on roundtable discussions and interviews highlighted low awareness of MSHT as a significant barrier, affecting both individuals with disabilities and professionals [5]. To address this gap, we used the AFPI tool to identify performance challenges clients were interested in addressing, which could be facilitated by MSHT, even if they had no previous knowledge of these technologies. This tool streamlined the task prioritization process. Although participants were informed that they could suggest tasks beyond the AFPI task bank, only a few additional tasks were proposed. Second, we used a demo station to facilitate device trials, focusing on selecting a suitable smart home ecosystem. Trialing mainstream smart home ecosystems is crucial, as they offer limited customization compared with traditional AT products. The hands-on experience provided participants with new information and helped them make more informed choices. For example, 2 participants, despite owning Amazon Echo devices, preferred Google Home after testing both systems. They found Google Home’s simple interface and well-spaced control buttons easier to navigate and use for selecting smart home device controls. In addition, they appreciated the seamless integration with the hands-free Google Assistant on their Android (Google LLC) phones. Similarly, another 2 participants who owned Amazon Echo devices expressed interest in also using Apple Home, as they were already using Siri (Apple Inc) for other tasks on their iPhones.

It was encouraging to find that 76% (13/17) of the participants in this study already owned at least 1 MSHT device. However, their attitudes toward technology, as reflected in the descriptors they selected, revealed mixed experiences. While 5 (29%) participants selected only positive descriptors, such as “satisfied,” “confident,” or “enjoyment,” most (n=12, 71%) participants chose either negative or a mix of descriptors. “Frustration” emerged as the most frequently selected negative descriptor, chosen by 9 (53%) participants. This aligns with our observation that, despite owning MSHT devices, some (3/17, 18%) participants had stored them away due to difficulties during setup, and most (10/17, 59%) were unsure how to integrate their devices into a smart home ecosystem. These findings suggest a need for targeted support to bridge the gap between ownership and effective use of MSHT, especially among populations with diverse abilities and needs.

It is also worth noting that more than half (10/17, 59%) of the participants in the study were enrolled in the US Medicaid home- and community-based services (HCBSs). Although the HCBS waiver definitions in the state of Pennsylvania include provisions for MSHT, such as electronic communication aids; mobile apps; hubs; software; and devices that control appliances, lights, telephones, and security systems, none of the participants were aware that they could request MSHT through their service providers. Furthermore, our investigation revealed a lack of clear processes for the provision of such technologies under the AT services in HCBS waiver programs.

This finding emphasized the need for better education and guidance for both service providers and participants to ensure these technologies are effectively integrated into care plans.

### Barriers to Implementation

The intervention faced several challenges, including factors related to participants, technology, training, and context.

#### Lack of Digital Literacy and Accessibility Knowledge

Some (7/17, 41%) participants faced challenges with digital literacy and accessibility, which created significant barriers to implementing MSHT effectively. These limitations impacted participants’ ability to interact with the technology, requiring study OTs to address these issues as part of the training. For example, 12% (2/17) of the participants were taught to use Amazon Alexa by clearing clutter around the Amazon Echo speaker, enabling audio cues, and pausing after the wake word to confirm the device’s responsiveness. OTs also provided guidance on password management, application organization, and accessibility features, such as voice control settings and voice assistants for participants relying on voice commands, as well as assistive touch settings for those needing improved touch interaction. While the MDPQ-16 assessed mobile device proficiency, it did not account for physical abilities or appropriate phone mounting on wheelchairs or bedside setups, which are critical factors for successful MSHT implementation for individuals with complex physical disabilities.

#### Technology Selection, Installation, and Customization

Selecting, installing, and customizing MSHT presented various challenges and often required significant time and effort. In some cases, it was difficult to find technology that fully met participants’ needs, as noted in the Results section, and in other instances, the technology only partially addressed their requirements. Device installation posed additional barriers. While Best Buy Geek Squad provided valuable services, it did not carry all the products provided to participants, necessitating the involvement of a general contractor. However, finding contractors knowledgeable about MSHT proved challenging. Beyond installation, pairing and integrating devices required substantial effort and often took longer than anticipated due to pairing complexities. Customizing solutions to meet participants’ unique needs added another layer of complexity. For example, one participant with both motor and visual impairments required a solution to respond to visitor calls and grant building access. To address this, we adapted a smart control button, linking it with the Tasker application (Crafty Apps Ltd) on his Android device. The button was configured for easier activation using his elbow and programmed via Tasker to simulate pressing “6” on his phone, enabling him to independently let visitors into the building. Another participant with a weaker voice experienced difficulties using a Fire TV Cube (Amazon.com, Inc) to control the television. This issue was resolved by adding a high-definition multimedia interface extension cable and an infrared blaster, positioning the device closer to the client to enhance speech recognition for voice commands.

#### Training

A significant barrier to implementation was the overlap between device setup and participant training during training sessions. The study OTs were tasked with pairing, configuring, and customizing devices to meet participants’ needs during the training sessions. The complexities of pairing for some devices and the time required for troubleshooting sometimes extended these sessions beyond 90 minutes. Ideally, all devices should be fully installed and configured before training begins to allow sessions to focus solely on skill development and effective use of the technology. This challenge highlights the need for a clear separation between device setup and training phases to streamline the implementation process.

#### Contextual Factors

Environmental and contextual limitations posed significant barriers to implementing MSHT. As noted in the Results section, environmental issues, such as missing electrical outlets, prevented the installation of devices needed to address participant needs. For 2 (12%) of the 17 participants living in subsidized housing, obtaining permission to install technology added delays and required additional effort, including writing justification letters and educating building contractors on MSHT installation. Caregivers also play a crucial role in sustaining the use of MSHT, particularly for devices requiring maintenance or caregiver collaboration. While caregivers were encouraged to participate in training sessions, their presence was not mandatory, which sometimes hindered effective implementation. For instance, 2 (12%) participants were provided similar technological solutions to manage their health care supplies, requiring caregivers to use a recommended tracking application; one participant found the solution effective for accurate tracking and timely ordering, while the other found it ineffective due to the caregiver not using the application to update supply information, highlighting caregiver engagement as a barrier.

In general, the findings of this study highlight significant opportunities to use MSHT to support a wide range of activities at a cost substantially lower than traditional AT, such as ECUs. MSHT offers versatility and affordability, making it a promising option for enhancing the independence and participation of individuals with complex physical disabilities. However, MSHT alone is not a complete solution. These individuals and their families often require ongoing support, including education on the available options, assistance with device selection, proper installation and setup, and training to ensure users feel confident and empowered. As emphasized in the Global Accessibility Reporting Initiative report [31], the concept of AT in the digital world must evolve to integrate mainstream technology into AT provision and include training on how to use these devices, their features, and associated services for professionals, caregivers, and end users with disabilities. Without the support, barriers such as lack of digital literacy, difficulty with setup, and underuse of features can limit the effectiveness of MSHT. Addressing these gaps is essential to ensuring that MSHT fulfills its promise of providing cost-effective, inclusive solutions for individuals with disabilities.

### Limitations

The study had several limitations. First, it reported only the quantitative results from the ASSIST intervention and excluded the 3-month follow-up data intended to evaluate continued use, performance, and satisfaction with MSHT after 3 months of independent use. As the study is ongoing, we chose to focus this paper on the immediate outcomes of the intervention due to the substantial volume of information collected, with plans to publish a subsequent paper addressing sustained use, qualitative feedback, and caregiver perspectives.

Second, the study primarily focused on participants with disabilities, with limited emphasis on caregiver involvement. While MSHT has the potential to significantly support caregivers, this aspect was not fully explored. Only 2 caregivers were enrolled in the study, and their experiences will be detailed in the follow-up paper. Future research should prioritize caregiver recruitment to comprehensively examine the dual impact of MSHT on individuals with disabilities and their caregivers.

Third, the study focused on participants with complex physical disabilities and had a small sample size. Future research should include a larger, more diverse sample to better assess the broader applicability of MSHT interventions.

Fourth, this study focused on commercial smart home technologies and did not evaluate open-source options for free alternatives. Further research should explore cost-effective options to enhance accessibility for individuals with disabilities.

Fifth, the intervention provided funding for MSHT devices and installation services to ensure a focus on evaluating their impact. However, this approach does not reflect real-world practices, where financial and logistical barriers can significantly affect access and use. Future studies should explore strategies for addressing funding and installation barriers in practical, real-world settings. In addition, the cost analysis did not account for clinicians’ time spent on device setup or delivering the intervention. A more comprehensive cost-benefit analysis would provide greater insights into the feasibility and sustainability of such interventions.

Finally, the study used a single-group pre-post design, which limits the ability to draw definitive conclusions about the causal effects of the intervention and understand the effects of its various components. A future comparative effectiveness study could incorporate a control group receiving either minimal support or specific subsets of the intervention components. This approach would enable researchers to evaluate the impact of individual elements within the intervention and identify which ones are most critical to achieving successful outcomes while still ensuring all participants derive some benefit. In addition, the treating OTs collected intervention outcomes, such as AFPI and self-perceived performance or satisfaction, which could bias responses due to social desirability or reluctance to give negative feedback. Future studies should use independent evaluators and observation-based tools to ensure objectivity and reduce bias.

### Conclusions

This study demonstrated the immediate benefits of the ASSIST intervention in promoting functional independence and improving satisfaction with MSHT among individuals with complex physical disabilities. The findings showed MSHT’s potential to address diverse daily living needs at a lower cost than traditional AT. However, challenges such as digital literacy and accessibility gaps, device setup barriers, and limited caregiver involvement present significant obstacles to broader implementation. Future work should prioritize overcoming these barriers by developing scalable models that incorporate funding solutions, caregiver engagement, and enhanced training programs. This study laid a strong foundation for advancing MSHT integration into the lives of individuals with disabilities and emphasized the need for a comprehensive, tailored approach to maximize its benefits and address implementation challenges.

## Abbreviations

ADL: activities of daily living
AFPI: Autonomy, Safety, and Social Integration via Smart Technologies Functional Performance Index
AI: artificial intelligence
ASSIST: Autonomy, Safety, and Social Integration via Smart Technologies
AT: assistive technology
ECU: environmental control unit
GAS: Goal Attainment Scale
HCBS: home- and community-based service
IADL: instrumental activities of daily living
IoT: Internet of Things
MDPQ: Mobile Device Proficiency Questionnaire
MSHT: mainstream smart home technology
OT: occupational therapist
REDCap: Research Electronic Data Capture
